# Bringing Art to Life: Measuring Engagement Through Art, Connection, and Dignity

**DOI:** 10.1093/geroni/igaa057.3054

**Published:** 2020-12-16

**Authors:** Candice Reel, Rebecca Allen, Bailey Lanai, Sami Sneed, Daniel Potts

**Affiliations:** 1 The University of Alabama, Tuscaloosa, Alabama, United States; 2 Alabama Research Institute on Aging, Tuscaloosa, Alabama, United States; 3 Tuscaloosa VA Medical Center, Tuscaloosa, Alabama, United States

## Abstract

The BATL program aims to improve the quality of life for community-dwelling people living with dementia (PWD) through art therapy, intergenerational contact, and life story preservation. This study utilized a modified ENGAGE measure (Hartmann et. al, 2017) to evaluate ethnographic transcripts recording activity and social engagement over time (N=100). A five-member analysis team independently coded the observation transcripts for engagement and qualitative themes. Results reveal average communication engagements (M=28.30, SD=13.36) significantly exceed the average art engagements (M=9.86, SD=5.56) over time t (98) = 20.85, p<0.001). Emergent themes included reminiscence, intergenerational communication, and creating art. These mixed method results suggest the intergenerational contact and reminiscence portions of BATL were fundamental features in comparison with art-making. More studies are needed to determine if the BATL intervention 1) has greater comparative effectiveness than typical programs in adult service centers, and 2) is scalable nationally to enhance the quality of life in PWD.

